# Causality of Aging Hallmarks

**DOI:** 10.14336/AD.2025.0541

**Published:** 2026-05-17

**Authors:** Bilu Huang, Xiaowen Hu

**Affiliations:** Fuzhuang Therapeutics Co., Ltd., Shanghai 200433, China

**Keywords:** aging, hallmarks, cause of aging, aging results, telomere, rDNA, P53

## Abstract

This article emphasizes the causal relationship in the mechanisms of aging, asserting that among the twelve hallmarks of aging, only telomere shortening is the cause of aging. The “Telomere DNA and ribosomal DNA co-regulation model for cell senescence” suggests that the shortening of telomeres and rDNA arrays can mediate various hallmarks of aging through the P53 pathway. Therefore, the best way to reverse aging and significantly extend lifespan is to increase the length of telomeres and rDNA arrays in adult stem cells within tissues.

## 1 Introduction

We all gradually age and eventually die, and many age-related diseases are also caused by aging. Therefore, instead of addressing each disease separately, it would be more effective to focus on solving the problem of aging itself. To solve the problem of aging, it is necessary to identify the root causes of aging. However, many changes associated with aging have been observed and touted as the driving forces behind aging [1]. In 2023, a review titled "Hallmarks of aging: An expanding universe" published in Cell listed the twelve major hallmarks of aging [2], but the authors did not provide causal arguments for each hallmark. This is because interventions targeting the consequences of aging are not very effective, whereas only those targeting the causes can significantly extend lifespan.

## 2 The Fundamental Cause of Cellular Senescence

Interventions targeting aging at the metabolic level and through signaling pathways have limitations: they result in only modest increases in lifespan, come with significant side effects, and are incapable of reversing aging. Therefore, we must first explore the fundamental causes of cellular senescence here, so that we can subsequently provide causal arguments for the various aging hallmarks.

### 2.1 The essence of aging is a program.

Since every organism has a relatively fixed timetable for growth, development, maturation, aging, and death, the essence of aging is a genetic program. Moreover, the program at the individual level is regulated by the program at the DNA level. For example, in the liver, hepatocytes express alpha-fetoprotein (AFP) during the fetal period, albumin during adulthood, and gradually express other proteins in old age [3]. Therefore, from the beginning of life as a fertilized egg, the gene expression profile can generally be divided into three modes: early, middle, and late. The early gene expression profile is mainly related to embryonic development. The gene expression pattern of cancer cells is fixed at this stage. For example, AFP, which is only expressed in fetal liver cells, is re-expressed when adult liver cells become cancerous. The middle gene expression profile is mainly related to maintaining health and reproduction. The late gene expression profile is mainly associated with the disruption of normal physiological functions. Therefore, the late gene expression pattern is the cause of degenerative diseases such as Alzheimer's disease, atherosclerosis, and hypertension [4]. Thus, the switching of gene expression patterns may be the main reason for the significant non-linear changes in individual aging. Glutathione, an endogenous antioxidant, remains almost unchanged before the age of 45, but begins to show a significant non-linear decline after 45 [5], thereby promoting aging and increasing protein oxidative damage, affecting protein fluidity and promoting the slow development of degenerative diseases [6].

After a long period of evolution, life has developed defense systems to overcome various types of damage. Therefore, even after sexual maturity, aging is not the result of the gradual accumulation of randomly occurring damage. Take the short-lived African killifish as an example [7], which can illustrate that aging after sexual maturity is still under programmatic control: The eggs laid by killifish will enter dormancy during the dry season and hatch again when ponds form with the arrival of the rainy season. In Zimbabwe, where the rainy season is brief, the puddles dry up quickly after the rain, and the local killifish strain (*Nothobranchius furzeri*) has a lifespan of only three months, equivalent to the length of the rainy season. In Mozambique, the rainy season is four times longer than that in Zimbabwe, and the local killifish strain (*Nothobranchius rachovii*) can live for nine months. In Tanzania, the local killifish strain (*Nothobranchius guentheri*) lives in an area with two rainy seasons and can have a lifespan of up to 16 months. When these three types of killifish are raised under the same conditions in captivity, the differences in their lifespans still persist [8]. This indicates that aging is controlled by a program, because the accumulation of random damage cannot explain why the lifespans of these three killifish strains, which have very similar body structures and belong to the same genus, differ so greatly and correspond exactly to the length of the rainy season.

If aging is controlled by a program, why is there a significant difference in lifespan within the same species or among identical twins? The answer is that the operating rate of the genetic program can be influenced by various factors that affect the rate of chemical reactions.

### 2.2 The manifestations of aging are the gradual decline and changes in function.

With increasing age, the synthesis rates of total cellular proteins and ATP will gradually decrease. Some proteins are specifically upregulated or downregulated, which are referred to as differentially expressed genes (DEGs). This leads to a gradual decline in the efficiency of cells performing various physiological functions and changes in function [9]. The downregulation of AFP and upregulation of albumin in mature hepatocytes represents functional changes. This is a kind of program that regulates individual growth, development, maturation, aging, and death.

There are two reasons for the decline in the total rate of protein synthesis in cells: (1) First, the decline in the total rate of protein synthesis is caused by chromatin condensation, as chromatin condensation is not conducive to DNA transcription. Chromatin condensation is associated with histone hypoacetylation. The level of histone acetylation in the liver of aging mice decreases, affecting hepatocyte regeneration [10]; (2) Since transcription factors are also proteins, with the decline in the total rate of protein synthesis in cells, the synthesis rate of most transcription factors will also decrease, thereby leading to a decline in the total rate of protein synthesis.

The tumor suppressor protein P53 shows a clear positive correlation with histone deacetylase (HDAC2) at the transcriptional level. Using the “P53 Signaling Super Path” in the PathCards database (http://pathcards.genecards.org/), it is found that many proteins of the HDAC family are involved in the P53-related SuperPath. This suggests that with the gradual upregulation of P53 levels due to cellular aging, the overall acetylation levels of histones H3 and H4 will also gradually decrease, leading to a gradual decline in the rate of DNA transcription, and consequently, a decline in the overall rate of protein synthesis.

### 2.3 The nucleus is the site that determines cellular aging.

In 1958, Yoshida immersed young leaves of *Elodea densa* in a 0.2 mol/L CaCl₂ solution, causing plasmolysis in the mesophyll cells. In some cells, the protoplast was divided into two parts, one containing the nucleus and the other without it. After 8 hours of observation under a microscope, it was found that the chloroplasts in the protoplast with the nucleus had gone through the process of aging and structural degradation, while the chloroplasts in the protoplast without the nucleus remained green and continued to accumulate starch [11].

In 1965, Wright and Hayflick transplanted nuclei from young cells into enucleated old cytoplasm. As a result, the cells regained their youthful state and continued to divide as many times as a young nucleus would. Conversely, when nuclei from old cells were transplanted into enucleated young cytoplasm, the cells exhibited aging phenotypes, and the number of cell divisions was significantly reduced.

These two experiments demonstrate that the nucleus is the site that determines cellular aging. Therefore, the various aging-related changes in the cytoplasm are not the causes of cellular aging, but rather the results mediated by the aging nucleus. In other words, mitochondrial dysfunction in the cytoplasm is merely a result mediated by the aging nucleus. A young nucleus can restore the normal function of dysfunctional mitochondria. Conversely, an aging nucleus can induce dysfunction in young mitochondria.

### 2.4 Cellular aging is co-regulated by telomeric DNA and ribosomal DNA.

Telomeric DNA and ribosomal DNA (rDNA) both belong to multicopy tandem repeat DNA arrays, which are highly unstable and prone to copy loss due to frequent transcription and other factors. For decades, it has been suggested that telomeric DNA and rDNA are associated with aging. However, the mechanisms by which these two types of DNA contribute to aging have not been unified, and it remains unclear whether the shortening of telomeric and rDNA arrays is a cause of cellular aging or merely a consequence of it. Moreover, many studies have proposed that excessively short telomeres or rDNA arrays induce cellular aging by triggering genomic instability and DNA damage. This explanation is clearly flawed. The residual lengths of telomeres and rDNA arrays in aging cells within the body are not short enough to cause genomic instability. Additionally, it raises the question of why cells gradually transition from a youthful to an aged state rather than aging abruptly.

There is substantial evidence indicating that telomeres are not the sole factor limiting cell division and aging. This is because in humans, many cells do not experience telomere shortening, yet their division capacity remains finite [12]. Additionally, maintaining longer telomeres through telomerase activity still fails to prevent replicative senescence [13-14]. Furthermore, the telomeres of yeast, nematodes, and fruit flies do not shorten. In some birds, telomeres even lengthen with an increasing age [15]. Therefore, in addition to telomeres, there must be another telomere-like entity that jointly regulates cellular aging. The “Telomere DNA and ribosomal DNA co-regulation model for cell senescence” proposes [16] that the fundamental cause of cellular aging is mediated by telomeric DNA and ribosomal DNA (rDNA) in the cell nucleus through the P53 pathway.

All types of aging cells upregulate P53, and the P53/P21/P16 signaling pathway plays an important role in regulating aging [17]. Approximately half of tumor cells have mutations in the *p53* gene, and deletion of the *p53* gene can enable cells to proliferate indefinitely [18], indicating that P53 is a master regulator of cellular aging. Therefore, various markers or phenotypes associated with aging are likely mediated directly or indirectly by P53.

By knocking down the copy number of 45S rDNA in mouse and human primary cells, the results showed that the aging markers P53, P21, P16, and SA-β-GAL were significantly upregulated, while telomere length, cell viability, and the number of cell passages were significantly reduced. Additionally, aging cells and human embryonic stem cells (hESCs) and human induced pluripotent stem cells (hiPSCs) from mice were examined. It was found that both telomere length and 45S rDNA copy number were significantly decreased in aging cells, whereas they were significantly increased in hESCs and hiPSCs. These data strongly demonstrate that the fundamental cause of cellular aging is regulated by both telomeres and 45S rDNA, and that the weight of rDNA in aging is greater than that of telomeres (unpublished observations).

**Figure 1.**
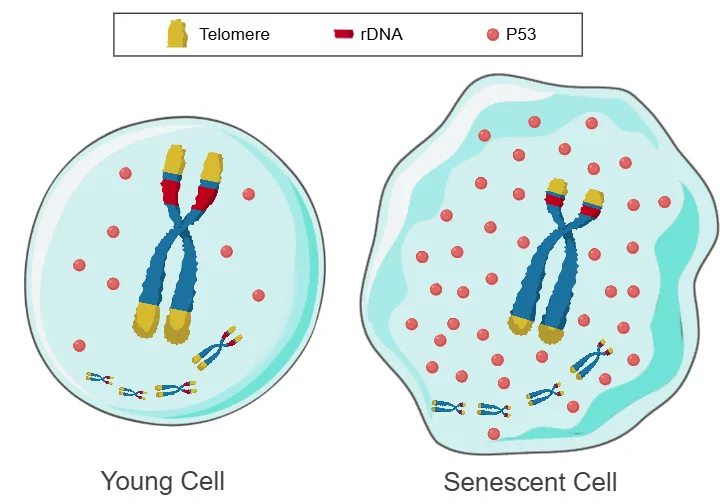
**Telomere DNA and ribosomal DNA co-regulation model for cell senescence.** Left: Chromosomes with long arrays of telomeres and rDNA: P53 is rapidly degraded, P53 levels are low, and the cell is youthful. Right: Chromosomes with short arrays of telomeres and rDNA: P53 is slowly degraded, P53 levels are high, and the cell is aged.

Human 45S rDNA is located between the short arms and satellite regions of the acrocentric chromosomes 13, 14, 15, 21, and 22. Acrocentric chromosomes refer to chromosomes with the centromere positioned near the end [19]. P53 is distributed at locations such as telomeres and rDNA [20-21], and it maintains a balance of P53 levels through degradation mechanisms. Compared to aging cells, young cells have longer telomeres and rDNA arrays and can rapidly degrade P53, keeping P53 levels low. Therefore, as the arrays of telomeric DNA and rDNA in cells gradually shorten, P53 levels will gradually increase, causing cells to transition from a youthful state to an aged state (Fig. 1).

### 2.5 rDNA also regulates the aging of non-dividing cells.

Although the aging of most dividing cells is co-regulated by telomeres and rDNA, in some terminally differentiated cells of mammals, somatic cells of worms, and fruit flies, chromosomal DNA is usually not replicated, making it less likely for telomeres to shorten. To ensure that these terminal cells also gradually age, there must be another mechanism to increase the levels of P53 in cells. For example, in fruit flies with a lifespan of 40 days, the rDNA array is reduced by half [22]. Theoretically, if rDNA were only for synthesizing rRNA, it could be multicopy but not necessarily tandemly repeated. However, given the high instability of rDNA, its multicopy tandem repeats, its ability to bind with the longevity protein Sir2 and the master aging regulator P53, and the fact that rDNA transcription easily leads to copy loss, the structure and properties of rDNA appear to be specifically designed to function as a second “counter” for controlling cell division and aging.

### 2.6 How do genes achieve programmed expression?

The process of development and aging is a process of programmed gene expression. However, in both young and aging cells, the sequence order and copy number of various genes on the chromosomes typically do not change over time. So, what force drives the fixed genes to undergo programmed expression (sequential expression) along the timeline?

Approximately 1/10 of human gene promoters contain P53 binding sites and can therefore be classified as P53-responsive genes [23]. P53 not only downregulates the overall rate of protein synthesis but also acts as a transcription factor that can simultaneously silence and activate numerous genes. Therefore, as the levels of P53 gradually increase, the overall rate of protein synthesis will gradually decrease, and a subset of genes will be specifically upregulated or downregulated. This allows cells to exhibit programmed gene expression while their function declines. Since different types of differentiated cells have distinct genetic programs, there are different gene expression patterns during the aging process. For example, in aging hematopoietic stem cells, the expression of approximately 1,500 genes undergoes significant upregulation or downregulation with increasing age [24].

Thus, telomeres and rDNA can be considered as indexes, while the genome functions as a database. Within the same type of differentiated cells, different lengths of telomeres and rDNA arrays result in different gene expression profiles. For instance, as telomeres gradually shorten, the expression activity of DUX4 can be upregulated by up to 10-fold [25]. Telomeres can influence gene expression by altering the epigenetic landscape [26]. Different lengths of rDNA arrays have varying distributions of heterochromatin/euchromatin, thereby changing the gene expression profile [27-28]. P53 can also directly or indirectly affect DNA (cytosine-5)-methyltransferase 3A (DNMTs). For example, P53 can bind to the promoter of the DNMT1 gene [29]. Therefore, as P53 levels change with age, the epigenetic patterns and gene expression profiles are altered. DNMT3A levels decrease with age, and hematopoietic stem cells deficient in this gene show increased or decreased methylation in different chromosomal regions, thereby changing the gene expression profile [30].

The key elements of a program are the clock, instructions, sequence, driving mechanism, and operational outcomes. Based on these elements, it is hypothesized that the operating mechanism of the gene program is as follows: As the telomeric DNA array and/or the rDNA array (the clock) shorten, P53 (the primary instruction) generates a "concentration gradient" along the timeline. Since P53 can bind to the promoters and enhancers of multiple genes (the sequence), it affects the expression of various genes, including those encoding transcription factors (secondary instructions) (the driving mechanism). This leads to the upregulation of some genes and the downregulation of others (the operational outcome).

### 2.7 Telomeric DNA and rDNA are the best candidates for the materials that drive the operation of genetic programs.

Although many genes can influence the rate of cellular aging and the levels of aging markers, they are not the fundamental cause of aging. Overall, to enable the fixed genes to achieve programmed expression, a timing device is needed to drive the process. Multicopy tandem repeat sequences of telomeric DNA and rDNA arrays fully meet all the requirements of a timer: (1) They can replicate a copy during cell division to ensure that each daughter cell has one; (2) The length of the array that shortens in somatic cells is replenished in germ cells, and the storage must be the highest in all cells of an individual to meet the consumption from embryonic development to maturation, aging, and death [31, 22]; (3) The rate of array shortening is flexible to accommodate the different developmental and aging rates of different cell types within the same species and across different species; (4) In somatic cells, telomeric DNA or/and rDNA can only be consumed unidirectionally to allow P53 to generate a "concentration gradient" along the timeline, forming a "measure of time," which enables genes to be expressed in a programmed manner to regulate an individual's growth, development, maturation, aging, and death; (5) Various external factors that affect the rate of aging, or the genes themselves, achieve their effects by influencing the rate of array shortening; (6) Since the lifespan of an individual can exceed a century, for example, the Greenland shark can live up to 400 years, the candidate material for driving the operation of genetic programs (equivalent to the sand in an hourglass) must be highly stable, without a half-life. Proteins, RNA, mitochondrial DNA (mtDNA), and chemical modifications of DNA and histones are all unstable, have half-lives, and are in a dynamic equilibrium of continuous degradation and replenishment. For instance, DNA methylation and demethylation, as well as histone acetylation and deacetylation, occur simultaneously. Therefore, they cannot form a measure of time and do not possess the attribute of timing. In other words, the fundamental cause of aging does not lie in RNA, proteins, mtDNA, or epigenetic modifications. Research in these directions will never uncover the fundamental cause of aging.

Whether a theory is correct depends on whether it is self-consistent. If telomeric DNA and rDNA arrays are indeed the driving forces for the programmed expression of gene clusters, then the shortened telomeric DNA and rDNA in somatic cells [32] must be replenished in the early embryonic cells or germ cells; otherwise, life would not be able to continue through generations. In other words, the arrays of telomeric DNA and rDNA are like the sand in an "hourglass timer"—once the sand runs out, it needs to be refilled. Fortunately, evidence already exists that the shortened telomeric DNA and rDNA arrays in somatic cells can be replenished in early embryonic cells or germ cells [31, 22]. It has also been found that the rejuvenation mechanism of human embryonic stem cells (hESCs) and human induced pluripotent stem cells (hiPSCs) is not due to epigenetic reprogramming, but rather because the lengths of the telomeric DNA arrays and the 45S rDNA arrays have significantly increased (unpublished observations). Therefore, the "Telomere DNA and ribosomal DNA co-regulation model for cell senescence" is the only highly credible theory of cellular aging.

### 2.8 Why is the telomere clock less accurate than the epigenetic clock in measuring physiological age?

As age increases, the overall level of DNA methylation across the genome gradually declines, based on which an epigenetic clock for measuring aging can be artificially defined [33]. In terms of accuracy, the epigenetic clock is more precise than the telomere clock. As for why the telomere clock is less accurate than the epigenetic clock in measuring physiological age, there may be the following three reasons:

1. Telomeres can regulate epigenetics [26], but the process from telomeres to epigenetics is relatively long and can be influenced by various factors along the way;
2. In humans, telomere length in tissues such as the testes, ovaries, cerebellum, vagina, skeletal muscle, and thyroid does not significantly shorten with increasing age [12]. The somatic cells of adult fruit flies also do not experience telomere shortening. The Brandt's bat (*Myotis brandti*), which weighs only 7 grams, has a lifespan of about 40 years, and its telomeres do not shorten [34];
3. Although it has been found that telomere and rDNA array shortening occur at the same level [32], telomere shortening can lead to rDNA array instability, and rDNA array instability can in turn promote shortening of the rDNA array. This explains why telomere and rDNA array shortening occur at the same level. For example, telomere disruption in human cells has been shown to immediately cause large-scale nucleolar defects, followed by rDNA array instability [35]. However, this is not always the case. The rate of telomere shortening starts off quickly, then gradually slows down [36-37], and eventually begins to rebound. For instance, telomere length in men and women slightly increases at ages 80 to 90 [38-39]. In naked mole-rats, telomeres actually lengthen with age [40]. This counterintuitive phenomenon may be due to telomeres compensating for rDNA array shortening. This has also been proven by knocking out rDNA copy numbers in aging cells, which leads to an increase in leukocyte telomere length (unpublished observations).

### 2.9 Individual aging is caused by the "replicative senescence" of adult stem cells.

Extracellular matrix components and misfolded or cross-linked proteins within cells are capable of being degraded and renewed. The accumulation of these waste materials in aging tissues occurs because the genetic program has shut down defense mechanisms. Therefore, individual aging cannot be caused by the accumulation of macromolecular waste.

Resident stem cells have been identified in all tissues and organs except the heart. These stem cells maintain tissue and organ homeostasis and repair through self-renewal and differentiation into new stem cells and "functional cells." However, both stem cells and functional cells can be eliminated by the immune system due to cellular senescence, genetic mutations, or viral infections. To compensate for these losses, stem cells must undergo repeated mitotic divisions. Since the number of divisions that adult stem cells can undergo is finite, and each division results in cells that are somewhat older than their predecessors, this leads to replicative senescence. Functional cells derived from senescent stem cells are also senescent, contributing to the aging of tissues, organs, and ultimately the individual. Therefore, the fundamental cause of individual aging ultimately stems from the replicative senescence of adult stem cells themselves.

If adult stem cells did not undergo replicative senescence, then the cellular senescence caused by factors such as DNA damage, cytotoxic compounds, and oncogene induction, and the resulting depletion of adult stem cells and functional cells, could all be replenished by adult stem cells through self-renewal and differentiation. In other words, if adult stem cells did not experience replicative senescence, individuals could remain youthful indefinitely.

As mentioned above, replicative senescence is caused by the shortening of telomeres and rDNA arrays. For example, in mouse hematopoietic stem cells, the shortening of the rDNA array is caused by the activation of mTOR1 [41]. The anti-aging drug rapamycin can inhibit cell replication and slow down replicative senescence by suppressing mTOR1.

In 1998, I published a monograph titled "The Mechanism, Significance, and Treatment of Aging" in "YANJING MEDICAL CORRESPONDENCE," in which I pointed out that the fundamental cause of individual aging is the shortening of telomeres and other repetitive DNA arrays in stem cells within tissues, rather than a reduction in the number of stem cells. I also ruled out factors such as mutations in nuclear DNA and mtDNA, which appear to cause cellular aging. Ionizing radiation can significantly induce mutations in both nuclear DNA and mtDNA. However, ionizing radiation does not shorten the lifespan of mice; on the contrary, it extends their lifespan. This important finding can also rule out the hypothesis that aging is caused by DNA mutations [42]. This is because, during the long process of evolution, organisms have developed redundant solutions to overcome these issues, which are gradually shut down by the genetic program. For example, mutations in mtDNA can be addressed through pathways such as "mitophagy" [43], so the accumulation of mutated mtDNA is merely a consequence of aging. Similarly, the accumulation of mutated DNA in the nuclei of aging cells occurs because the DNA repair system and the immune system for clearing senescent and mutated cells are progressively shut down. Therefore, the theory that mutations in nuclear DNA and mtDNA in aging cells are the cause of individual aging cannot explain the vast differences in lifespan, ranging from the less than 20-day lifespan of the nematode Caenorhabditis elegans to the over 400-year lifespan of the Greenland shark.

Regarding the argument about the relationship between individual aging and stem cells, in 2007, Anastasia et al. from the UK published a paper in the journal Nature stating that stem cells are crucial for human self-repair and tissue regeneration, and that the reduction in the number of stem cells is the main cause of human aging [44]. The conclusion of Anastasia et al. is incorrect. First, it is not that there are not enough stem cells in the human body, but rather that there is a system controlling the number of stem cells. For example, in the scalps of balding patients, hair follicle stem cells are still present, but they are unable to grow hair [45]. In old mice, the number of hematopoietic stem cells increases by five times [46]. Additionally, the human body has an immune system that can clear DNA damage. Therefore, the aging of the immune system is the main culprit for the accumulation of DNA-mutated cells in tissues, and the aging of the immune system is a derivative result of the aging of hematopoietic stem cells. This is because the various immune cells that are replenished through the differentiation of aged hematopoietic stem cells are also aged immune cells.

In 2010, Sahin and DePinho from the US also published a paper in Nature, stating that the main cause of individual aging is the shortening of telomeres and mtDNA mutations in tissue stem cells [47]. The conclusion of Sahin and DePinho is also incorrect. First, mutated mtDNA can be selectively cleared through pathways such as "mitophagy" [43]. Second, cells with mitochondrial dysfunction cannot compete with healthy cells and are eliminated. It has also been found that mouse models with increased mtDNA mutations show no signs of accelerated aging and do not have shortened lifespans. Heterozygous mutations in superoxide dismutase lead to increased oxidative damage and mtDNA mutations but do not shorten the lifespan of mice.

In theory, replacing the aged adult stem cells in aging tissues with young adult stem cells could rejuvenate the aged tissues. However, transplanted allogeneic stem cells will be cleared by the immune system. Transplanting autologous stem cells that have been expanded in vitro is also problematic, as the expansion process induces replicative senescence. Additionally, transplanting adult stem cells derived from induced pluripotent stem cells (iPSCs) generated from autologous cells is not viable, because these cells often harbor detectable DNA damage exceeding 70% [48], which leads to their immune clearance [49]. Therefore, these approaches are not feasible. The only viable solution is to increase the length of telomeres and rDNA arrays in autologous adult stem cells.

It is generally believed that the human body has 78 organs. However, histologists consider each bone to be an organ (and there are also several subtypes of skeletal stem cells [50]), which means the human body actually has a total of 284 organs. Moreover, the hair follicle stem cells in hair, eyebrows, eyelashes, nasal hair, armpit hair, pubic hair, and body hair are not all identical. Therefore, considering the various subtypes of adult stem cells, there may be more than 300 types of adult stem cells in the human body. As a result, transplanting just a few types of young adult stem cells cannot completely rejuvenate an aging individual.

## 3 Causality of 12 Aging Hallmarks

**Figure 2.**
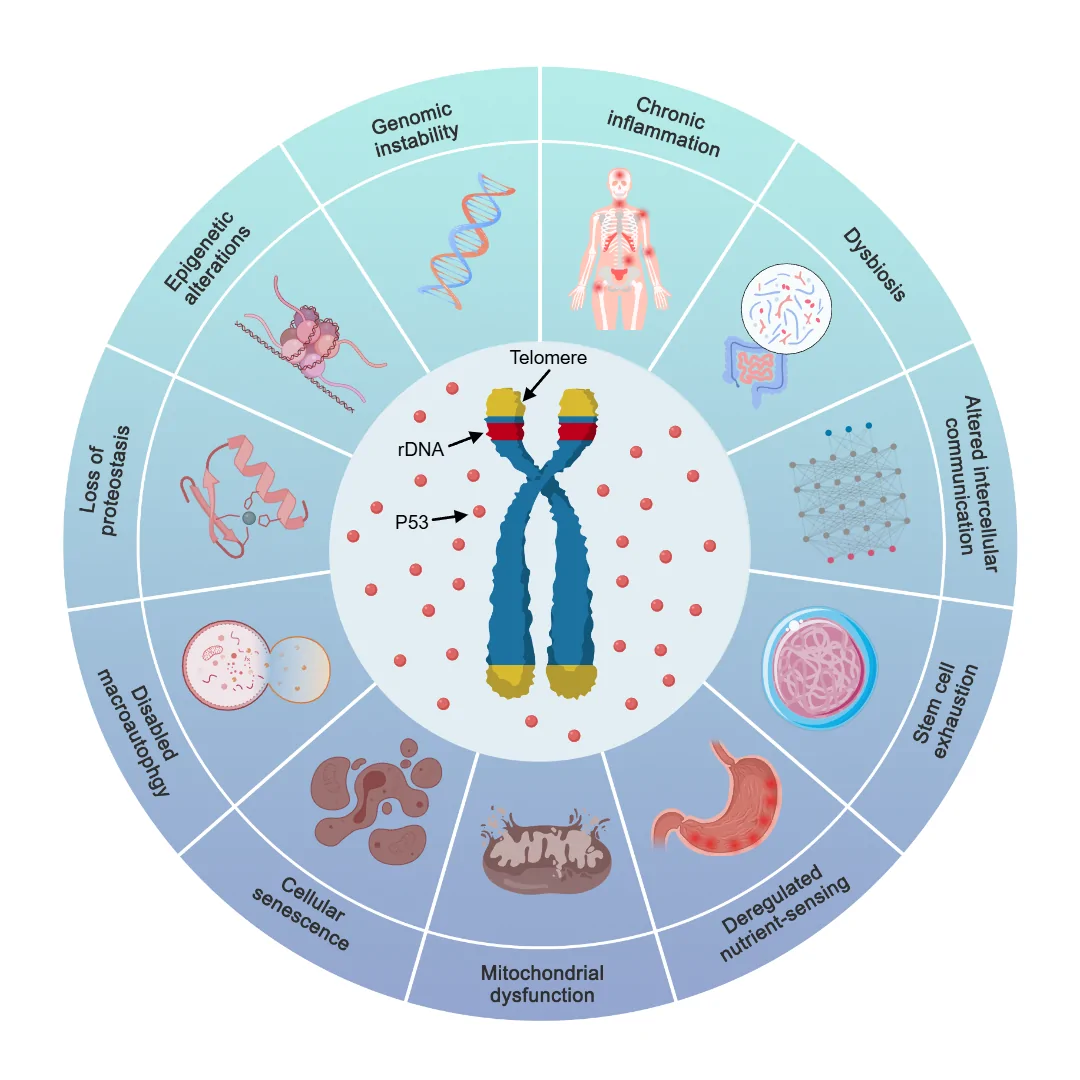
The eleven major hallmarks of aging mediated by telomeres and rDNA through P53.

Many people believe that it may take decades to clarify the causal relationships of aging [51]. Since P53 is the master regulator of aging, we will next explore the causal relationships between the twelve major hallmarks of aging and P53 (Fig. 2).

### 3.1 Genomic instability

As age increases, the genome becomes increasingly unstable, making chromosomes more prone to aberrations, DNA more susceptible to mutations, and leading to the activation of endogenous retroviruses (ERVs), which are normally silenced repetitive elements.

So, what causes genomic instability?

Telomeres or rDNA arrays that are too short can lead to genomic instability and trigger a DNA damage response [52]. However, the initial telomere lengths in mice and humans are 50 kb and 15 kb, respectively, and the telomere shortening rate in mice is 6,420 bp per year, which is 100 times faster than that in humans [53]. Therefore, the remaining telomeres in aged mice are still longer than the initial telomeres in humans, and thus genomic instability is unlikely to occur. In fact, telomeres and rDNA arrays in human aging cells still retain half of their initial length, so the genomic instability observed in aging cells is not related to the physical length of telomeres and rDNA arrays.

The DNA in heterochromatin regions is in a state of hypermethylation and histone hypoacetylation, which allows histones to tightly wrap around the DNA and maintain genomic stability. DNA methyltransferase DNMT1 plays a crucial role in maintaining the hypermethylation state of heterochromatin DNA.

P53 can bind to the promoter of the DNMT1 gene [29]. Therefore, as the arrays of telomeres and rDNA gradually shorten, the progressively upregulated P53 leads to a gradual decrease in the expression of the DNMT1 gene. This reduces the methylation levels of DNA in heterochromatin regions, resulting in "loss of heterochromatin" and genomic instability.

Genomic instability is associated with the functional inactivation of centromeres and is one of the important causes of chromosome segregation errors. The function of centromeres relies on a specific histone variant, CENP-A. In aging cells, CENP-A is downregulated in a P53-dependent manner [54].

SIRTs can bind to telomeres and rDNA to maintain the stability of these regions. However, P53 can inhibit the expression of all seven SIRT proteins, thereby leading to instability in telomeres and rDNA regions within the nucleolus. In yeast, Sir2 binds to the rDNA region [55], and Sir2, together with Sir3 and Sir4, binds to telomeres [56-57]. In mammals, SIRT7 is localized to the nucleolus, where it maintains rDNA stability [58].

The loss of heterochromatin can lead to the transcription of silenced endogenous retroviruses (ERVs). Since P53 can also activate the transcription of ERVs [59], this results in sterile chronic inflammation. Chronic inflammation is believed to promote aging [60]. However, treatment with reverse transcriptase inhibitors (NRTIs) has not been observed to extend the lifespan of wild-type mice [61], indicating that the release of ERVs is merely a consequence of aging.

Heterochromatin generally contains high-density repetitive DNA elements, such as telomeric sequences, satellite sequences, rDNA repeat sequences, and transposable elements. Therefore, heterochromatin is primarily located in centromeric, telomeric, and nucleolar regions [62]. As mentioned above, telomeric DNA and rDNA are both multicopy tandem repeat arrays, which are highly unstable and prone to copy loss due to frequent transcription and other factors. Therefore, increasing genomic stability can slow the shortening of telomere and rDNA arrays [16, 26].

Based on the "Telomere DNA and ribosomal DNA co-regulation model for cell senescence" [16], it is hypothesized that the differences in lifespan among species depend on the rate of shortening of telomere and rDNA arrays. It has been observed that the shortening rates of telomeres and the rDNA arrays in the nucleolus are at the same level [32]. Caloric restriction (CR) and rapamycin both delay aging by inhibiting mTOR1, which in turn suppresses the transcription of 45S rDNA in the nucleolus, thereby reducing the loss of 45S rDNA copies. The following examples also support the idea that inhibiting the transcription of 45S rDNA can delay aging:

The anti-aging drug spermidine can significantly downregulate the activity of mTORC1 within cells, thereby inhibiting rDNA transcription [63]; RPL22 can promote the transcription of 45S rDNA and induce cellular aging by disrupting the heterochromatin structure in the nucleolus [64]; Mice with 4EBP1 gene knockout activate mTORC1, promote the transcription of 45S rDNA, and accelerate cardiac aging [65]; There is evidence that eating less can lead to a longer lifespan. Overfeeding fruit flies excessively stimulates mTOR1, thereby accelerating the shortening of the 45S rDNA array [66]; In mouse hematopoietic stem cells, the 45S rDNA array shortens with the activation of mTOR1 [41]; IL-11 upregulates the ERK-AMPK-mTORC1 axis, and its levels increase with age in mice. Inhibiting IL-11 with antibodies can extend the lifespan of mice [67].

Compared to young mice, aged mice accumulate more DNA mutations. DNA mutations are not a cause of cellular aging but rather a result of the aging immune system, which is unable to effectively monitor and clear mutated cells. Studies have also found that DNA mutations do not lead to cellular aging [68]. The fact that killifish can accumulate a large number of DNA-mutated cells and develop cancer within less than six months [69] also illustrates that the accumulation of DNA-mutated cells is due to the aging of the immune system. Since the accumulation of DNA damage is merely a consequence of the aging immune system, it cannot explain the vast differences in lifespan between species such as the short-lived Caenorhabditis elegans, which lives for only a little over 10 days, and the long-lived Greenland shark, which can live for over 400 years. Similarly, it cannot account for the significant lifespan differences within the human body, such as the short-lived cells that live for only a few days and the cardiomyocytes which can live for a century.

Tumor cells possess genomic instability yet can achieve immortality, and epigenetic age is not affected by radiation-induced DNA breaks and the resulting genomic instability [70]. This indicates that cellular aging is not caused by genomic instability.

### 3.2 Telomere Attrition

The length of telomeric DNA arrays shortens due to chromosomal DNA replication, which is one of the fundamental causes of cellular replicative senescence. This is because lengthening telomeres can significantly increase the number of cell divisions, reduce aging markers [71], decrease DNA damage and cancer incidence [72], and extend the median lifespan of mice by 40%, along with a return of coat color [73]. However, in addition to telomeres, rDNA is also a major regulator of cellular aging [16], and according to our observations, the impact of rDNA on aging is greater than that of telomeres. Therefore, telomere shortening is not only a marker of aging but also a cause in the causal relationship of aging.

Telomere length is negatively correlated with DUX4 gene expression [25]. DUX4 is negatively correlated with MHC-I gene expression. Therefore, as telomeres continuously shorten, DUX4 expression gradually increases, while MHC-I expression gradually decreases. Consequently, the upregulation of DUX4 affects MHC antigen presentation [74], which is one of the reasons for the accumulation of senescent cells in aging tissues and the higher incidence of cancer in middle-aged and older adults compared to younger individuals. Moreover, the true reason that telomere shortening increases cancer susceptibility is not chromosomal instability, but rather that shorter telomeres lead to T-cell immune deficiencies [75]. In Ashkenazi Jewish centenarians, mutations in hTERT and hTERC are associated with longer telomeres, longer lifespans, and protection against age-related diseases [76]. The cancer incidence rate in the group with the shortest telomeres is three times higher than that in the group with the longest telomeres, and the mortality rate from cancer in the group with the shortest telomeres is 11 times higher than that in the group with the longest telomeres [77].

Overall, increasing telomere length can rejuvenate the immune system, thereby reducing the accumulation of senescent cells in tissues and the incidence of tumors.

### 3.3 Epigenetic Alterations

As age increases, epigenetic features such as DNA methylation patterns and histone modifications undergo changes, which affect gene expression and cellular functions.

However, age-related epigenetic changes are also regulated by telomeres and rDNA [26-28]. P53 can bind to the promoter of the DNMT1 gene to mediate epigenetics [29]. Therefore, epigenetic changes are not the cause of aging but rather a result of the shortening of telomere and rDNA arrays. Thus, anti-aging approaches that solely target epigenetic modifications are not viable. For example, partial reprogramming (expressing Oct4, Sox2, Klf4, and C-myc) in 12-month-old wild-type mice did not extend lifespan [78]. The median lifespan of partially reprogrammed wild-type mice only increased by 12%, which is less effective than small-molecule anti-aging drugs [79]. The slight increase in lifespan may be related to the cell regeneration-promoting properties of the Yamanaka factors Oct4, Sox2, Klf4, and doxycycline used in reprogramming. For instance, promoting angiogenesis can significantly extend the mouse lifespan [80]. After partial reprogramming was completed, it was found that telomeres did not lengthen or were slightly shortened, aging markers began to accumulate again, and the epigenetic age returned to the state before reprogramming [81].

Growth hormone can reverse the DNA methylation clock [82], but it shortens the lifespan of mice. Rudman's growth hormone supplementation experiment in elderly individuals found that growth hormone could increase muscle mass and thicken the skin in individuals aged 60-80, making them appear 10-20 years younger. However, follow-up studies revealed that elderly individuals who received long-term growth hormone supplementation not only failed to extend their lifespan but also experienced accelerated aging. A 60-year-old individual who received gene therapy for growth hormone-releasing hormone (GHRH) showed a reversal in physiological age, with a 28.6-year reduction in PhenoAge clock age and a 6-year reduction in epigenetic age, but their telomere age was 7 months older than that of peers [83].

The paradox of growth hormone reducing epigenetic age while shortening lifespan is essentially about promoting protein and ATP synthesis. When tested, it shows a younger state, but in reality, it accelerates cell division, overdrawing the limited telomere and rDNA arrays, or in other words, overdrawing the limited number of cell divisions. It's like a candle: the bigger the flame, the faster it burns. Many anti-aging measures actually overdraft the limited number of cell replications. Instead of achieving rejuvenation, they may lead to a shortened lifespan.

### 3.4 Loss of Proteostasis

As age increases, tissue cells accumulate misfolded and mistranslated proteins, which in turn affect cellular functions.

The decline in gene expression levels of heat shock protein HSP70 is a major cause of the loss of protein homeostasis. Monocytes from the 65- and 85-year-old age groups have significantly lower HSP70 expression levels compared to the 25-year-old age group [84].

The Interventions Testing Program (ITP) (www.nia.nih.gov/research/dab/interventions-testing-program-itp), the most rigorous aging intervention test by the National Institute on Aging, has tested the effects of various compounds on lifespan in a genetically heterogeneous mouse model (UM-HET3). Geranylgeranylacetone (GGA) has been shown to induce the expression of heat shock proteins in mammalian tissues, ensuring that proteins fold correctly. However, ITP testing found that GGA does not extend mouse lifespan [85], indicating that the age-related loss of proteostasis is not a cause of cellular aging but rather a consequence of it.

P53 can bind to the promoter of the SIRT1 gene, thereby inhibiting the transcription of SIRT1 [86]. SIRT1 promotes the transcription of heat shock protein HSP70 [87]. Therefore, the shortening of telomere and rDNA arrays, which leads to upregulation of P53 levels, suppresses the expression of the SIRT1 gene, thereby reducing HSP70 levels and increasing the accumulation of misfolded proteins.

### 3.5 Disabled Macroautophagy

Autophagy is tasked with clearing abnormal proteins and organelles to maintain cellular health. However, this mechanism becomes less efficient as age increases.

Knockout or pharmacological inhibition of P53 can induce autophagy in human, mouse, and nematode cells [88]. Researchers have questioned the “consensus” that autophagy promotes longevity [89]: Data from studies on worms and mammals show that the permeability of mitochondria determines the impact of autophagy on aging. When mitochondrial permeability is increased, enhancing autophagy can actually be detrimental to health. Ovarian aging in mice is associated with increased autophagy in granulosa cells [90]. Chloroquine (CQ) can inhibit autophagy. Treating wild-type aging rats with chloroquine for five months extended their median lifespan by 6% and maximum lifespan by 13%, which is comparable to some of the best anti-aging compounds tested in mouse models to date [91]. Enhancing autophagy can promote health in young worms but also accelerates the aging process. Inhibiting autophagy in aged worms leads to significant improvements in neuronal and overall health, with lifespan extended by as much as 50% [92]. Therefore, spermidine and rapamycin, which are known to enhance autophagy, do not extend lifespan by enhancing autophagy. Instead, they extend lifespan by inhibiting mTOR1 to downregulate rDNA transcription, thereby reducing rDNA array shortening.

The often-contradictory effects of enhancing autophagy on lifespan suggest that autophagy dysfunction is not a cause of aging, but rather a consequence of aging.

### 3.6 Deregulated Nutrient-sensing

The development of aging and metabolic diseases is closely related to the regulation of nutrient sensing (such as AMPK and SIRT1) [93], and the capacity for nutrient sensing is downregulated with aging.

As mentioned above, the shortening of telomeres and rDNA arrays leads to an upregulation of P53 levels, and the upregulation of P53 in turn results in a downregulation of overall protein synthesis rates and specific upregulation or downregulation of certain genes. Therefore, the downregulation and alteration of the synthesis rates of various receptors and ligand proteins related to nutrient sensing are the main reasons for the downregulation of nutrient sensing with increasing age. Consequently, mice with extra-long telomeres exhibit greater tolerance to insulin and glucose, lower cholesterol and low-density lipoprotein levels, and a leaner body shape [72].

Peroxisome proliferator-activated receptor-γ coactivator-1α (PGC-1α) is a key transcription factor that plays a role in glucose homeostasis and has a significant impact on energy balance and the fundamental pathways of diabetes. During the aging process, the levels of PGC-1α decline, leading to health problems [94]. SIRT1, through its deacetylation of PGC-1α, can prevent the proteasomal degradation of PGC-1α. In contrast, P53 promotes the degradation of PGC-1α by inhibiting the expression of SIRT1. As a result, aged mice are unable to maintain plasma glucose levels during fasting [95].

### 3.7 Mitochondrial Dysfunction

Mitochondria are the powerhouses of the cell. As age increases, ATP production gradually declines, affecting cellular energy supply and health status.

When worms were supplemented with the antioxidant vitamin C, mitochondrial ATP production increased by more than 2.5 times. On the 8th day after treatment, the content of lipofuscin, a type of age pigment, increased by about 18%, and their lifespan was significantly shortened. In contrast, doxycycline, which inhibits ATP production, extended the lifespan of young worms by 72.8% when administered at a concentration of 13 µM (6 μg/ml). On the 13th day after treatment, the content of lipofuscin decreased by about 50% [96]. Doxycycline also extends the lifespan of progeroid mice [97].

Short telomeres lead to mitochondrial dysfunction [98]. Among the 50 nuclear genes encoding mitochondrial proteins, the expression of 25 genes is significantly positively correlated with rDNA dosage [99]. Mouse models with extra-long telomeres also exhibit better mitochondrial function and less DNA damage [72]. As mentioned above, P53 inhibits the expression of PGC-1α and PGC-1β. The downregulation of PGC-1α/β expression, in turn, leads to mitochondrial dysfunction and reduced ATP production.

Heterozygous mutations in the mouse mitochondrial superoxide dismutase (SOD2) lead to increased oxidative damage and mitochondrial DNA (mtDNA) mutations, but do not shorten lifespan [100]. Compared to wild-type mice, mice with 50 times more mtDNA mutations show no signs of premature aging, such as osteoporosis, alopecia, or reduced fertility [101]. To date, no reports have demonstrated that enhancing mitochondrial function can effectively extend lifespan, suggesting that mitochondrial dysfunction is a consequence of aging rather than a cause.

### 3.8 Cellular Senescence

Cellular senescence contributes to the aging of tissues, organs, and the entire organism. Senescent cells are cells that can no longer divide but secrete inflammatory factors. The accumulation of senescent cells can affect the renewal and repair of tissues.

In 2011, Baker DJ et al. administered a drug that could clear senescent cells to wild-type aged mice. Although their health improved, their lifespan did not increase [102]. Female mice that began taking dasatinib and quercetin to clear senescent cells from a young age actually experienced accelerated aging [103]. In humans, the clearance of senescent cells using dasatinib and quercetin was observed to significantly increase epigenetic age while significantly shortening telomere length [104]. Treatment with ABT-263 to clear senescent cells accelerated ovarian aging in aged female mice [105]. This is because the clearance of senescent cells stimulates the replication and differentiation of surrounding younger cells to fill the void left by the removed senescent cells, thereby inducing replicative senescence.

### 3.9 Stem Cell Exhaustion

Resident adult stem cells have been identified in all tissues and organs except the heart, and they are responsible for the renewal and repair of tissue cells. Therefore, adult stem cells need to divide repeatedly. However, the number of times adult stem cells can replicate is limited, and each replication results in a cell that is slightly older than its predecessor, thereby inducing replicative senescence. For example, although the number of NK cells increases with age, their efficiency in clearing tumor cells decreases significantly. This is one of the important reasons why the elderly are more prone to cancer [106]. In the scalps of balding patients, hair follicle stem cells are still present, but they are unable to grow hair [45]. The number of hematopoietic stem cells in aged mice increases fivefold [46].

Overall, stem cell exhaustion is not related to a decrease in the number of stem cells, but rather to the replicative senescence of the stem cells themselves. The resident adult stem cells in tissues and organs, due to repeated divisions, experience continuous shortening of telomeres and rDNA arrays, which in turn lead to the gradual upregulation of P53, resulting in replicative senescence. Therefore, the shortening of telomeres and rDNA arrays in adult stem cells is the fundamental cause of individual aging.

### 3.10 Altered Intercellular Communication

Cell-to-cell communication is essential for maintaining tissue function, and this communication is disrupted during the aging process.

The process of cellular senescence is a gradual alteration of the gene expression profile [107]. Different ages have distinct gene expression profiles. Therefore, the number and activity of receptors and ligands for various communication molecules also change with increasing age. Coupled with the interference from inflammatory signals produced by senescent cells, this ultimately leads to changes in cell-to-cell communication.

### 3.11 Chronic Inflammation

Aging is accompanied by an increase in chronic low-grade inflammation, which is associated with many age-related diseases.

Aging nuclei and mitochondria release DNA fragments into the cytoplasm. The body mistakes this for a viral infection and produces a sterile chronic inflammation to summon immune cells to clear the senescent cells [60, 108].

Senescent cells produce a group of inflammatory factors, also known as the Senescence-associated Secretory Phenotype (SASP). P53 activates the downstream gene *p21*, which then promotes the production of SASP by reducing the phosphorylation of Rb [109]. P53 binds to the promoter of the inflammatory factor IL-22 gene to promote the transcription of the IL-22 gene [110]. Alzheimer's disease (AD) brain tissue is in an inflammatory state, with high levels of P53 in the cortical neurons of the AD brain and in the skin [111-113]. P53 can induce the expression of endogenous retroviral elements (ERVs) that should be silenced, triggering inflammation [59].

As an inflammatory factor, IL-11 is upregulated with increasing age in mice and activates the ERK-AMPK-mTORC1 signaling pathway. Inhibiting IL-11 with antibodies can extend the average lifespan of mice by more than 22% [67]. Since IL-11 upregulates the mTOR1 signaling pathway, the mechanism by which IL-11 inhibition extends mouse lifespan may not be due to reduced inflammation levels, but rather akin to taking the anti-aging drug rapamycin, an mTORC1 inhibitor. This is because macrophage migration inhibitory factor antagonists (MIF098) can also inhibit chronic inflammation in the body. The anti-aging drug NR, which increases NAD+, can reduce the release of inflammatory factors by 52.6% [114]. However, the ITP test revealed that NR actually shortened mouse lifespan by 3%. MIF098 had no effect on lifespan [115]. ERVs can trigger inflammation, but no study has found that reverse transcriptase inhibitors (NRTIs) can extend the lifespan of wild-type mice [61]. Compared to short-lived shark species, three gene families involved in activating the NF-kB signaling pathway (TNF, TLR, LRRFIP) are significantly increased in the Greenland shark. The authors suggest that the significant increase in inflammatory genes leads to enhanced immunity, which contributes to the extreme longevity of the Greenland shark [116]. This indicates that chronic inflammation is merely a consequence of aging, and efforts to delay aging by inhibiting inflammation have been declared unsuccessful.

P53 inhibits the expression of seven SIRT proteins, and the deficiency of SIRT3 exacerbates P53/Parkin-mediated inhibition of mitochondrial autophagy [117]. P53 inhibits Parkin-mediated mitochondrial autophagy and promotes mitochondrial dysfunction in the mouse heart [118]. When mitochondrial autophagy is impaired, mtDNA leaks from the mitochondria into the cytoplasm, activating the cGAS-STING pathway and triggering an inflammatory response [119]. Therefore, an increase in P53 levels inhibits mitochondrial autophagy, causing mtDNA leakage into the cytoplasm and inducing sterile inflammation.

### 3.12 Dysbiosis

This refers to the imbalance of the internal environment of the body, including changes in the microbiota, which can affect our health.

Transplanting the gut microbiota from young killifish to aged killifish can extend the lifespan of the aged killifish [120]. However, before feeding the young killifish gut bacteria, antibiotics must first be used to kill the gut bacteria of the aged killifish. As mentioned above, doxycycline, which is used for partial reprogramming, can extend the lifespan of worms and mice. Therefore, the beneficial effects of antibiotics themselves could also extend the lifespan of aged killifish. Moreover, killifish have a very short lifespan, and the newly established microbiota may not have time to cause significant damage before the fish die. Thus, while young fecal microbiota can extend the lifespan of aged killifish, it may not necessarily extend the lifespan of mice, which have a longer lifespan, let alone human lifespan. Recently, there was a paradoxical and contradictory finding: gut microbiota from aged hosts had a beneficial lifespan-extending effect on young mice [121].

Restoring the gut microbiota of aged individuals to a youthful composition through fecal microbiota transplantation may not effectively reverse aging [122]; the uniqueness of the microbiota in long-lived populations may not be related to healthy aging [123]. The gut microbiota undergoes significant changes with increasing age [124]. Intestinal secretions, particularly bile acids, are important determinants of microbial abundance, diversity, and metabolic activity [125-126]. P53 regulates bile acid synthesis and thereby affects bile acid homeostasis by modulating the small heterodimer partner (SHP). P53 inhibits the expression of bile acid synthesis enzymes CYP7A1 and CYP8B1 through its response elements (P53REs) [127]. P53 can significantly upregulate the expression of enzyme genes involved in bile acid hydroxylation (such as CYP3A11 and CYP2B10) and sulfation (such as SULT2A1), while also promoting the expression of bile acid efflux transporters (such as ABCC2, ABCC3, and ABCC4) [128]. Therefore, the composition and levels of bile acids change with increasing age [129-130]. Therefore, in the absence of changes in dietary habits, the age-related changes in the composition of the gut microbiota are determined by the age-related changes in gastrointestinal secretions. This suggests that gut microbial dysbiosis is a consequence of individual aging, rather than being inherent to the microbes themselves.

The intestinal mucus layer acts as a barrier for the gut to prevent bacteria from entering the bloodstream. *Akkermansia muciniphila*, known as a health supplement, is a bacterium that feeds on the mucin in the intestinal mucus layer. When normal wild-type mice were orally administered *Akkermansia muciniphila*, the number of *Citrobacter rodentium* in their feces increased significantly. *Citrobacter rodentium* not only broke through the intestinal barrier but also entered the bloodstream, causing systemic infection and leading to severe pathological damage in the colonic tissue [131].

The Russian Nobel Prize laureate Ilya Ilyich Mechnikov (1845-1916) believed that consuming yogurt to alter the gut microbiota could significantly extend lifespan. He himself drank yogurt daily in his later years and lived to the age of 71. However, subsequent surveys and statistical analyses have shown that people who drink yogurt are not necessarily more long-lived than those who do not.

In summary, the 11 aging hallmarks mediated by P53 are shown in Table 1.

**Table 1:** The 11 Hallmarks of Aging Mediated by Telomere and rDNA Array Shortening through P53.

Aging Hallmark	The Hallmarks of Aging Mediated by Telomere and rDNA Array Shortening through P53	References
Genomic instability	P53 mediates the expression of DNMT1 and CENP-A genes, thereby affecting the methylation levels of DNA in heterochromatin regions and the function of centromeres, leading to genomic instability.	Peterson et al. [29], Sikder et al. [54]
Epigenetic alterations	P53 mediates the expression of the DNMT1 gene, affecting epigenetics.	Peterson et al. [29]
Loss of proteostasis	P53 affects the transcription of heat shock protein HSP70 mediated by the SIRT1 gene, thereby affecting proteostasis.	Nemoto et al. [86], Brunquell et al. [87]
Disabled macroautophagy	P53 mediates autophagy in human, mice, and nematode cells.	Tasdemir et al. [88]
Deregulated nutrient-sensing	P53 mediates PGC-1α and plasma glucose levels by affecting SIRT1 expression.	Sahin et al. [95]
Mitochondrial dysfunction	P53 suppresses the expression of PGC-1α and PGC-1β. Downregulation of PGC-1α/β expression leads to mitochondrial dysfunction and reduced ATP production.	Sahin et al. [95], Hoshino et al. [118]
Cellular senescence	P53 mediates histone acetylation levels and the expression of various transcription factor genes, thereby affecting protein synthesis rates and gene expression profiles.	http://pathcards.genecards.org/, Yang et al. [17]
Stem cell exhaustion	Stem cell exhaustion is characterized by reduced and altered stem cell function, mediated by P53 through decreased protein synthesis rates and altered gene expression profiles.	http://pathcards.genecards.org/, Yang et al. [17]
Altered intercellular communication	Different ages have distinct gene expression profiles, and thus the number and activity of receptors and ligands for various communication molecules change with increasing age.	Schaum et al. [107]
Chronic inflammation	Under the influence of P53, mitochondria and nuclei in senescent cells release mtDNA, dsRNA, and eccDNA into the cytoplasm. The body perceives this as a viral infection, leading to sterile chronic inflammation.	Zhou et al. [59], Liu et al. [60], Newman et al. [108]
Dysbiosis	Changes in bile acid levels mediated by P53 affect the structure of the gut microbiota.	Kim et al. [127], Chen et al. [128]

## 4 Discussion

As research continues, new biomarkers of aging will continue to emerge. Since aging markers are not the causes of aging, this process seems endless, much like the expanding universe. For example, a new biomarker of aging has been proposed: immunoglobulin IgG. IgG can directly induce the senescence of macrophages and microglia in humans and mice, leading to the release of inflammatory factors [132]. This is because the accumulation of useless waste can produce a hormetic effect, manifesting symptoms of aging. For instance, the accumulation of microplastics activates key inflammatory markers such as SA-β-galactosidase, NF-κB, IL6, TNF-α, and CD68, similar to other aging markers [133]. Recently, a review article increased the number of aging hallmarks from 12 to 14 [134].

In fact, many theories of aging and interventions are incorrect, and this preprint has specifically criticized them [135]. Overall, the current academic community is studying the mechanisms of aging at the protein and RNA levels. However, through logical deduction and experimental research, we believe that the driving substances determining cellular senescence, and the number of replications are telomere DNA and rDNA. In other words, the first tier of aging regulation is at the DNA level, and epigenetics and proteins are merely mediators of DNA-level regulation. To break through the ceiling of lifespan, it is essential to lengthen telomeres and rDNA arrays. Beyond this, simply reversing epigenetics or regulating signaling pathways, among other interventions, are futile. Therefore, in the future, the gold standard for various anti-aging interventions must include an increase in the length of telomeres and rDNA arrays. For lifespan control experiments in mice, wild-type mice must be used instead of progeroid mice.

In summary, the shortening of telomeres and rDNA arrays is the fundamental cause of cellular senescence. Therefore, anti-aging methods that target various aging biomarkers have very limited effects and cannot reverse aging. To truly reverse aging and significantly extend lifespan, the best approach is to increase the length of telomeres and rDNA arrays in adult stem cells within tissues.

## Data Availability

Not applicable.
